# *IlAP2*, an AP2/ERF Superfamily Gene, Mediates Cadmium Tolerance by Interacting with *IlMT2a* in *Iris lactea* var. *chinensis*

**DOI:** 10.3390/plants12040823

**Published:** 2023-02-12

**Authors:** Zhiquan Wang, Longjie Ni, Liangqin Liu, Haiyan Yuan, Chunsun Gu

**Affiliations:** 1 Institute of Botany, Jiangsu Province and Chinese Academy of Sciences (Nanjing Botanical Garden Memorial Sun Yat-Sen), Nanjing 210014, China; 2 Jiangsu Key Laboratory for the Research and Utilization of Plant Resources, Nanjing 210014, China; 3 College of Forest Sciences, Nanjing Forestry University, Nanjing 210037, China

**Keywords:** cadmium, *Iris lactea* var. chinensis, *IlAP2*, *IlMT2a*

## Abstract

Cadmium (Cd) stress has a major impact on ecosystems, so it is important to find suitable Cd-tolerant plants while elucidating the responsible molecular mechanism for phytoremediation to manage Cd soil contamination. *Iris lactea* var. *chinensis* is an ornamental perennial groundcover plant with strong tolerance to Cd. Previous studies found that *IlAP2*, an AP2/ERF superfamily gene, may be an interacting partner of the metallothionein gene *IlMT2a*, which plays a key role in Cd tolerance. To study the role of *IlAP2* in regulating Cd tolerance in *I. lactea*, we analyzed its regulation function and mechanism based on a yeast two-hybrid assay, a bimolecular fluorescence complementation test, quantitative real-time PCR, transgenics and transcriptome sequencing. The results showed that IlAP2 interacts with IlMT2a and may cooperate with other transcription factors to regulate genes involved in signal transduction and plant hormones, leading to reduced Cd toxicity by hindering Cd transport. These findings provide insights into the mechanism of *IlAP2*-mediated stress responses to Cd and important gene resources for improving plant stress tolerance in phytoremediation.

## 1. Introduction

Human activity, natural pollution such as volcanic ash, climate change and the use of recycled water, among other reasons, have resulted in an increase in the area of cadmium (Cd)-contaminated soil [1,2,3]. The presence of Cd in soil is a serious environmental hazard, as the metal is not only toxic to plants but also transmissible through the food chain, threatening ecosystems and human health [4,5]. Compared with physicochemical treatment, phytoremediation is an economical, natural and effective method for managing Cd soil contamination [6,7]. Therefore, it is important to find suitable Cd-tolerant plants and elucidate their relevant molecular mechanisms.

Cd toxicity can inhibit carbon fixation and reduce chlorophyll content and photosynthetic activity [8]. When plants are exposed to Cd-contaminated soil, osmotic stress occurs, reducing the relative moisture content, stomatal conductance and transpiration [9]. Meanwhile, an excessive occurrence of reactive oxygen species (ROS) can damage plant membranes and destroy cell biomolecules and organelles [10]. Cd also interferes with the transport and uptake of mineral elements [9]. To avoid the harm caused by Cd stress, plants have undergone a series of morphological, physiological and biochemical evolutionary changes [11]. One major mechanism is external exclusion, which prevents plant cells from absorbing excessive Cd and hinders Cd transport within the plant [12,13]. Another mechanism is tolerance to Cd accumulation, whereby Cd exists in a non-biologically active conjugated form within the plant through chelation and compartmentalization [14]. Studies have reported that when Cd is absorbed by a plant, proteins such as metallothioneins (MTs) can form stable chelates with the Cd ion, thus reducing its toxicity [4,15]. The regulation of transcription factors is a vital mechanism in plant cells that protects them from heavy metal exposure [16]. For example, previous studies showed that metal-responsive transcription factor 1 (MTF1) is involved in cellular protection against Cd stress signals [17]. Under Cd stress conditions, MTF1 can translocate into the nucleus and bind to metal-responsive elements (MREs), mediating the transcription of a series of downstream genes such as MTs, ZIP10, ferroportin 1 (FPN 1), selenoprotein H (Sel H) and selenoprotein W (Sel W) [16].

*Iris lactea* var. *chinensis*, an ornamental perennial groundcover plant that grows fast and forms a large biomass, is resistant to several stresses [18,19]. In particular, it has a very strong tolerance to Cd and the ability to accumulate Cd, which makes it suitable for improving Cd-contaminated soil [20]. We preliminarily analyzed the regulatory mechanism of *I. lactea* in response to Cd stress through transcriptome sequencing and mined an MT gene, *IlMT2a*, that may play a key role in Cd tolerance [21]. When constitutively expressed in *Arabidopsis thaliana*, *IlMT2a* leads to greater root length under Cd stress compared to the wild type (WT) [22]. In turn, we identified several proteins interacting with IlMT2a using a yeast two-hybrid assay (Y2H) with a yeast library constructed from cadmium-treated *I. lactea* seedlings and found that IlAP2, an apetala2/ethylene responsive factor, may be an interacting partner of IlMT2a [3].

Many transcription factors can be regulated by Cd stress and induce MTs to confer Cd tolerance [23,24,25], and *AP2* genes have been specifically induced by Cd in many plants, including *A. thaliana*, rice and kenaf [26,27,28]. Many reports have documented that AP2s are important regulators involved in plant growth and development, hormonal regulation, plant metabolite biosynthesis and the conferring of stress tolerance to plants [29,30]. Recently, potato *StAP2/ERF*s were found to be indispensable for Cd uptake and tolerance and may be useful in designing gene-modified plants with improved Cd tolerance [31]. To study the role of *IlAP2* in regulating the Cd tolerance of *I. lactea*, we cloned the open reading frame of *IlAP2* and confirmed the interaction between IlMT2a and IlAP2 using Y2H and a bimolecular fluorescence complementation test (BiFC). Following that, the regulation function and mechanism were explored through genetic transformation and transcriptome sequencing. This study provides information on the molecular mechanism of Cd tolerance and a gene resource for improving plant tolerance to Cd.

## 2. Results

### 2.1. Cloning and Analysis of the I1AP2 Sequence

**Figure 1:**
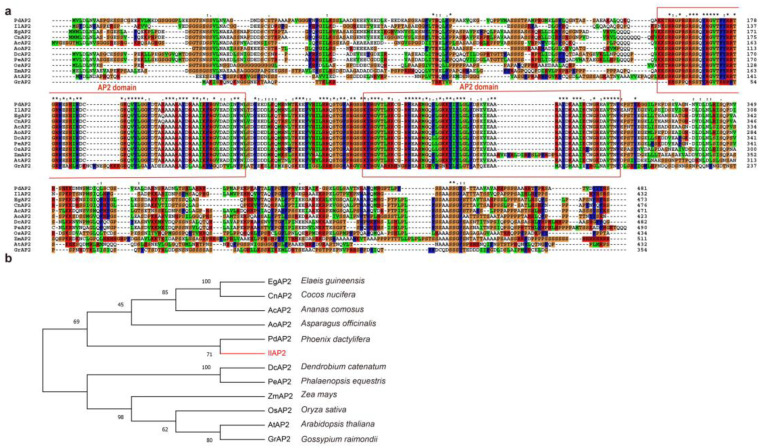
Analysis of the AP2 sequence: (**a**) alignment result of IlAP2 amino acid sequence (the red boxes represent functional domains, ’*’ indicates positions which have a single, fully conserved residue); and (**b**) phylogenetic tree of AP2 proteins from *I. lactea* (IlAP2) and other species.

Based on transcriptome data, primers were designed to obtain the full-length ORF sequence of IlAP2. The ORF of *IlAP2* was 1296 bp and encoded 432 amino acids. Several proteins with high similarity to IlAP2 in other plant species were selected using Blast on the NCBI website, and their amino acid sequences were analyzed using ClustalX software (Figure 1a). The alignment results showed that IlAP2 contained two conserved AP2 domains (Figure 1a). A phylogenetic tree was constructed using the neighbor-joining method to further analyze the evolutionary relationship between the *IlAP2* and *AP2* genes in other plants. The results showed that *IlAP2* was most closely related to the genes of *Phoenix dactylifera* (Figure 1b).

**Figure 2:**
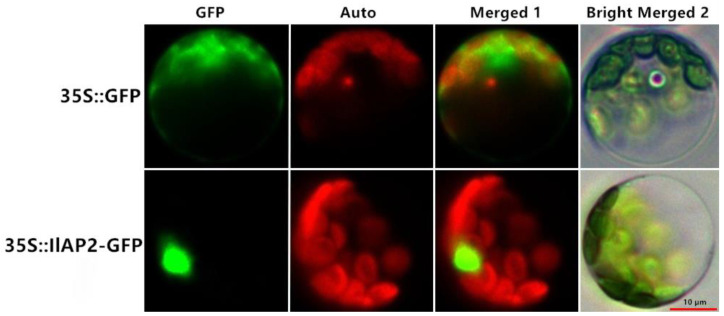
Subcellular localization of IlAP2 in *A. thaliana* leaf cells, with GFP as control. Green fluorescence protein (GFP), chlorophyll autofluorescence (Auto) and merged images (Merged 1 and Bright Merged 2) are shown. Scale bar: 10 μm.

To further understand the cytological function of the IlAP2 protein, we inserted *IlAP2* into a pCAMBIA expression vector with an eGFP tag and transgenically transformed *A. thaliana* protoplasts to observe the subcellular distribution of IlAP2 proteins. The *IlAP2*–GFP fusion protein was only expressed in the nucleus, while the empty GFP vector was expressed in all parts of the cell, which indicates that *IlAP2* is a nuclear-localized transcription factor (Figure 2).

### 2.2. Confirmation of Interaction between IlAP2 and IlMT2a

**Figure 3:**
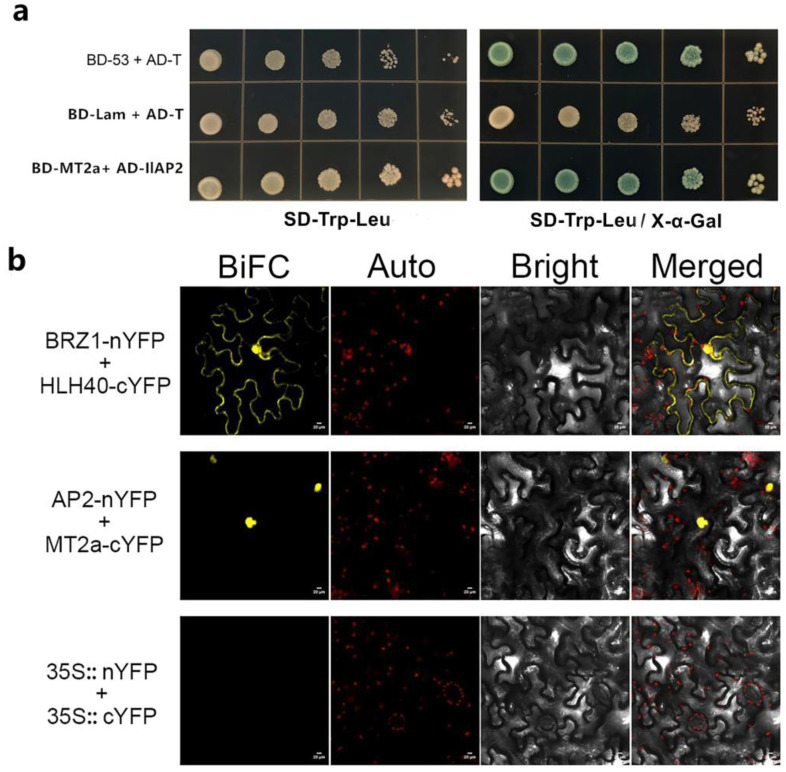
Interaction between IlAP2 and IlMT2a: (**a**) yeast two-hybrid analysis of IlAP2 and IlMT2a, with BD-53 + AD-T as positive control and BD-Lam + AD-T as negative control; and (**b**) BiFC analysis of IlAP2 and IlMT2a, with BRZ1-nTFP + HLH40-cYFP as positive control and 35S::nYFP + 35S::cYFP as negative control. Ruler: 20 μm.

We re-inserted the complete *IlAP2* sequence into a pGADT7 vector and verified the interaction between IlAP2 and IlMT2a by Y2H based on the screening results using SD-Trp-Leu-Ade-His (QDO plates) [3,32]. The positive control (BD-53 + AD-T), negative control (BD-Lam + AD-T) and yeast cells co-transformed with PGADT7–*IlAP2* and PGBKT7–*IlMT2a* all grew normally in the SD-Trp-Leu medium, while only the positive plasmid and yeast cells co-transformed with PGADT7–*IlAP2* and PGBKT7–*IlMT2a* turned blue in the SD-Trp-Leu/X-α-Gal medium, indicating that IlAP2 and IlMT2a interact in yeast (Figure 3a). In addition, we carried out a BiFC experiment. *A. tumefaciens* containing *IlAP2*–NYFP and *IlMT2a*–CYFP plasmids were co-injected into tobacco leaves. The obvious interaction between IlAP2 and IlMT2a was verified again. The BiFC fluorescence was located in the nuclear region (Figure 3b).

### 2.3. IlAP2 Expression Pattern Analysis

**Figure 4:**
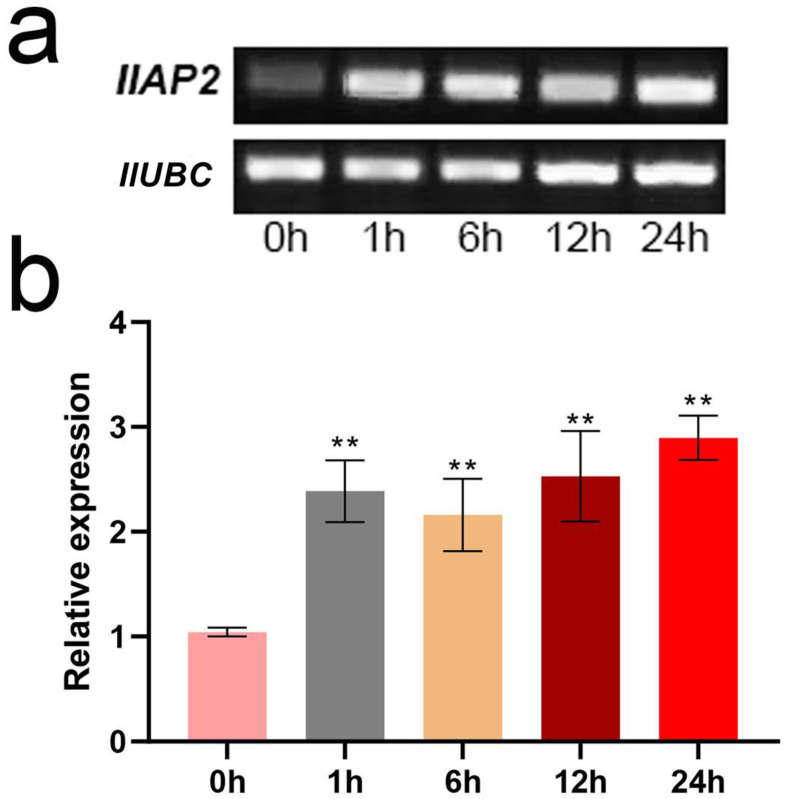
Analysis of *IlAP2* expression pattern in *I. lactea* roots at different times of Cd stress: (**a**) semi-quantitative PCR and (**b**) RT-qPCR. ‘**’ shows the significance level of *p* < 0.01.

To explore the expression pattern of *IlAP2* in response to Cd stress, semi-quantitative PCR and RT-qPCR were used to analyze the expression of *IlAP2* in *I. lactea* roots under 50 μM CdCl_2_ stress at different times. Semi-quantitative PCR showed up-regulated *IlAP2* expression after stress treatment, which was further demonstrated by RT-qPCR (Figure 4). There was no significant difference in *IlAP2* expression level among the different treatment times (Figure 4).

### 2.4. Functional Verification of I1AP2 in A. thaliana

**Figure 5:**
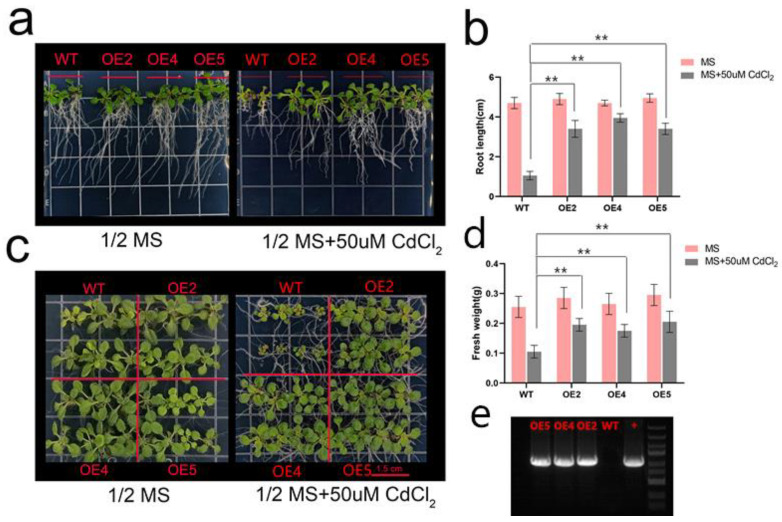
Functional verification analysis of *I1AP2* in *A. thaliana*. (**a**) Root length of wild-type (WT) and transgenic *A. thaliana* under CdCl_2_ stress. (**b**) Root length statistics of WT and transgenic *A. thaliana* under CdCl_2_ stress. (**c**) Comparison of growth of WT and transgenic *A. thaliana* under CdCl_2_ stress. (**d**) Fresh weight of WT and transgenic *A. thaliana* under CdCl_2_ stress. (**e**) Agarose gel electrophoresis profile for the expression of *I1AP2* in WT and transgenic *A. thaliana* (OE2, OE4 and OE5). The + band was amplified with *I. lactea* cDNA and ORF primers of *I1AP2*, serving as positive control. ‘**’ shows the significance level of *p* < 0.01.

To investigate whether *IlAP2* can improve Cd tolerance, we constructed *I1AP2*-overexpressing *A. thaliana* and obtained eight homozygous T3-generation transgenic lines. Three independent transgenic lines were selected for functional verification: OE2, OE4 and OE5. After 10 days of growth (3-day-old seedlings were exposed to cadmium for 7 days), there was no significant difference in root length and growth between the WT and transgenic plants grown on normal half-MS medium. Under a 7-day Cd treatment, although the root length and growth of the WT and transgenic plants decreased continuously, the degree of decline for the transgenic plants was slower than that of the WT plants (Figure 5), suggesting that *IlAP2* improved Cd tolerance in *A. thaliana*.

### 2.5. Analysis of the Potential Mechanism of IlAP2 under Cd Stress

**Figure 6:**
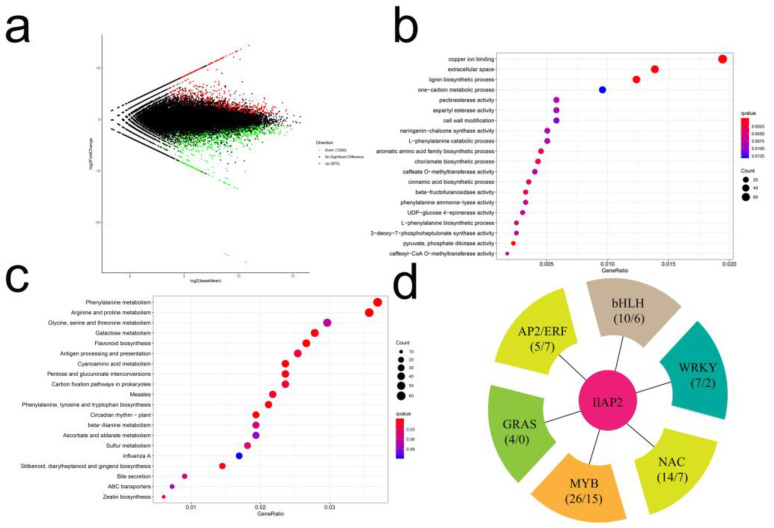
RNA-seq analysis of WT and transgenic *A. thaliana*. (**a**) DEG analysis results, (**b**) GO enrichment results, (**c**) KEGG enrichment results and (**d**) gene families possibly affected by *IlAP2*.

To further explore the mechanism by which *IlAP2* regulates Cd tolerance, RNA-seq analysis was performed on transgenic *A. thaliana* seedlings. The comparison between the transgenic *A. thaliana* and the WT identified 2210 genes as DEGs: 1340 up-regulated and 870 down-regulated (Figure 6a). The GO enrichment analysis showed that the enrichment degree of transferase activity was the highest in molecular function, indicating that ion signal transduction played an important role in *I. lactea*‘s response after 24 h of Cd stress (Figure 6b). The KEGG enrichment analysis showed that plant hormone signal transduction was significantly enriched (Figure 6c), suggesting that plant hormones also played an important role in response to Cd stress. In addition, we found that bHLH, WRKY, NAC, MYB, GRAS, AP2/ERF and other transcription factors were all affected by *IlAP2* after 24 h of Cd stress (Figure 6d). We randomly chose six differentially expressed transcription factors and analyzed the relative changes in expression using qRT-PCR (Figure S1). The results showed that the expression patterns of most of the transcription factors tested with qRT-PCR were similar to those obtained from the sequencing data. Additionally, it was proven again that the transcription factors were affected by *IlAP2*.

## 3. Discussion

Heavy metal pollution of soil and water is a serious environmental problem. Among heavy metal pollutants, Cd is considered to be one of the most toxic ones [24]. Cd can be quickly absorbed by plants due to its high solubility in water, which is the main way it enters the food chain [33]. Plant roots can absorb and transport Cd to nutritional and reproductive organs even at low Cd concentrations, which has a negative impact on the regulation of the plant’s nutrition and its mineral balance during growth [34].

Several genes in plants, such as those encoding an ATP-binding transporter, ZRT/IRT-like proteins and glutathione, have been reported to participate in reducing Cd ion toxicity by inhibiting Cd ion absorption and transport, improving Cd ion chelation ability and other processes [35,36]. Gene *BcMT2* could enhance Cd tolerance and reduce the production of reactive oxygen species (ROS) in *A. thaliana* [37]. Similarly, we previously discovered that *IlMT2a* in *I. lactea* increased plant tolerance to Cd by potentially reducing the accumulation of H_2_O_2_ and O_2_^•−^ [22]. However, *IlMT3*, another MT, was identified as an interacting protein of *IlMT2a* but did not provide protection against Cd toxicity in transgenic *A. thaliana* [3]. In this study, an AP2/ERF superfamily gene that encodes an interacting protein of IlMT2a, further validated its role in *I. lactea*‘s response to Cd stress.

The transformation of transcriptomics under Cd stress has been studied in several plant species, which has helped to identify many Cd-responding transcription factors [24]. Different transcription factors, such as ERF (ethylene-responsive factor), WRKY, bZIP (basic leucine zipper) and MYB (myeloblastosis protein), control the expression of specific stress-related genes under Cd stress and play important roles [24]. Based on the ORF sequence amplified by PCR, the gene was named *IlAP2* following the phylogenetic tree and amino acid sequence analyses. To further understand the cytological function of the IlAP2 protein, we studied its expression and found that it was localized in the nucleus. In addition, the BiFC assays showed that the site of interaction between *IlAP2* and *IlMT2a* was also in the nucleus. The expression pattern analysis indicated that *IlAP2* was a stress-responsive transcription factor that could be regulated by Cd stress and maintained a steady high level of expression under Cd stress treatment. These findings indicate that *IlAP2* may be involved in the response to Cd stress and has a positive effect on Cd-stress tolerance, similar to some *StAP2/ERF* genes in kenaf [23].

Plant-specific AP2/ERF transcription factors can regulate plant responses to environmental stimuli or plant growth and development, depending on the presence of a highly conserved 60-amino-acid AP2 domain in the protein [38,39]. For example, rice *OsEREBP1* attenuates disease caused by *Xanthomonas* and confers drought and submergence tolerance by activating the jasmonate and abscisic acid signaling pathways, thereby priming the rice plants for enhanced survival under abiotic or biotic stress conditions [40]. *A. thaliana RAP2.6* participates in abiotic stress, including abscisic acid (ABA), salt and osmotic stress, possibly through the ABA-dependent pathway [41]. A previous study found that *StAP2/ERF*s are indispensable in Cd uptake and tolerance and may be useful in designing gene-modified potato plants with improved Cd tolerance [31]. To further demonstrate that *IlAP2* can improve plant Cd tolerance, we performed phenotype analysis on overexpressing *A. thaliana* plants. Under normal conditions, the phenotypic difference between the transgenic and WT *A. thaliana* plants was not significant. After Cd treatment, the growth of all *A. thaliana* plants was inhibited, but the root length and growth of the WT were inhibited to a greater extent, indicating that *IlAP2* influences the response of *A. thaliana* to Cd stress.

The overexpression of *TdSHN1*, a wheat ERF transcription factor, in transgenic tobacco conferred Cd tolerance, produced less ROS under excess Cd compared to WT plants and had higher activities of ROS-scavenging enzymes, which might contribute to Cd tolerance [42]. IlAP2 was confirmed to interact with IlMT2a and improve Cd tolerance. Although there is no report on the overexpression of *AtAP2* in *Arabidopsis*, the expression of potato *StAP2/ERF075/077/126* has been shown to increase tolerance to Cd [31]. Therefore, *AP2* may play an important role in the regulation of cadmium tolerance. Based on these results, we used transgenic *Arabidopsis* to preliminarily explore the regulatory mechanism through transcriptome sequencing, laying the foundation for subsequent research. A total of 1,340 and 870 genes were identified as up- and down-regulated, respectively. Enrichment analysis showed that *IlAP2* could alter ion signal transduction, plant hormones and other transcription factors at the transcriptional level, playing an important role in the plant’s response to Cd stress. Previous studies have preliminarily analyzed the regulatory mechanism of *I. lactea* in response to Cd stress through proteome data and found that Cd regulatory proteins were mainly involved in signal transduction, ion transport, redox reactions, ion binding and other functions [43]. These findings indicate that *IlAP2* interacts with *IlMT2a* in the nucleus and may cooperate with other transcription factors to regulate genes related to signal transduction and plant hormones, thereby reducing Cd toxicity.

## 4. Materials and Methods

### 4.1. Plant Materials

The plant materials were preserved in Nanjing Botanical Garden Memorial Sun Yat-Sen. Selected seeds of *I. lactea* were cultivated under a photoperiod of 16/8 h (day/night) in a greenhouse incubator for 6 weeks. Seedlings about 10 cm high were washed and transferred to 50 mL centrifuge tubes containing 1/2 Hoagland solution (Table S1) for 1 week. Following that, the seedlings were transferred again to centrifuge tubes containing 1/2 Hoagland solution with 50 μM CdCl_2_, as described in a previous study [21]. The roots were sampled at time points of 0, 1, 3, 6, 12 and 24 h, with three biological replicates for each time point. Samples at 0 h served as controls. All sampled plant materials were immediately frozen in liquid nitrogen and stored at −80 °C.

Tobacco (*Nicotiana benthamiana*) and *A. thaliana* (ecotype Columbia) seeds were selected and sown in plastic pots containing mixed culture medium (peat, vermiculite and perlite, 1:1:1 by volume) after soaking in sterile water for 24 h. The leaves obtained were used for research on subcellular localization and BiFC testing. Transgenic and WT *A. thaliana* seeds were prepared and sterilized with 10% NaClO for 10 min, then sown in 1/2 Murashige and Skoog (1/2 MS) medium supplemented with 3% (*w*/*v*) sucrose and 0.8% agar, incubated in darkness at 4 °C for 24 h and grown in a sterile environment.

### 4.2. Sequence Alignment and Subcellular Localization

The open reading frame (ORF) was amplified using Takara LA Taq (Takara, Dalian, China) based on the sequence obtained from transcriptome data (Table 1), and the PCR product was purified and cloned into the pMD19-T vector (Takara) for sequencing verification. Protein sequence was used for alignment using ClustalW (v. 2.1) software, and a phylogenetic tree was constructed using MEGA 7.0 with the neighbor-joining method following 1000 bootstrap replications.

A ClonExpress II One Step Cloning Kit (Vazyme, Nanjing, China) was selected to construct the recombinant plasmid vector containing the target sequence without the stop codon and the pBI121–GFP vector. The recombinant plasmids were transformed into *A. thaliana* protoplasts, which were isolated with enzymatic hydrolysis [44,45] using the PEG transformation method. Confocal laser scanning microscopy (Zeiss LSM 710 META, Jena, Germany) was used to determine the localization of gene expression through observing GFP fluorescence [46].

### 4.3. Semi-Quantitative PCR and Quantitative Real-Time PCR (qRT-PCR)

Total RNA of *I. lactea* roots under Cd stress was extracted using Plant RNeasy Mini Kit (Vazyme) and cDNA was generated with PrimeScript^®^ RT kit (Takara, Dalian, China). Oligo 6.0 was used to design primers for semi-quantitative PCR and qRT-PCR (Table 1). Semi-quantitative PCR and qRT-PCR were performed using *IlUBC* as the reference gene [20]. Each reaction mix of semi-quantitative PCR contained 1 μL Taq enzyme, 2 μL 10 × PCR Buffer (Mg^2+^ Free), 1.5 μL Mg^2+^ (25 mmol·L^−1^), 1.3 μL dNTP mixture (2.5 mmol·L^−1^ each), 6 pmol of each primer and 1 μL cDNA (about 1000 ng·μL^−1^). PCR was performed under the following cycling conditions: 94 °C for 4 min, 25 cycles of 94 °C for 30 s, 55°C for 30 s, 72°C for 30 s and 72 °C for 10 min. PCR products were detected by 1% agarose gel electrophoresis. qRT-PCR was performed using AceQ qPCR SYBR Green Master Mix (Vazyme, Nanjing, China) and StepOnePlus real-time PCR system (Applied Biosystems, Foster City, CA, USA) according to the manufacturer’s instructions. The reaction mixture consisted of 2 µL diluted cDNA, 10 µL 2 × SYBR Green Master Mix (Bimake, Houston, TX, USA), 0.4 µL ROX (Dye I), 1 µL of each forward and reverse primer and ddH_2_O to a total volume of 20 µL. The PCR procedure included an initial denaturation step set at 95 °C for 10 min, followed by 40 cycles of 95°C for 15 s, 60 °C for 30 s and 72 °C for 30 s. Melting curve analyses of the amplified products were conducted at 60–95 °C. Three biological replicates were performed for each treatment and three technical replicates for each reaction. Expression levels were analyzed with the 2^−ΔΔCT^ calculation method.

### 4.4. Y2H and BiFC

The full-length ORF sequence of *IlAP2* was inserted into the PGADT7 vector using a ClonExpress II One Step Cloning Kit (Vazyme) to form the recombinant vector PGADT7–*IlAP2*. According to the Yeast Protocols Handbook (PT3024-1, Clontech, USA), the negative control (PGBKT7-LAM + PGADT7-T), positive control (PGBKT7-53 + PGADT7-T) and recombinant vector (PGADT7–*IlAP2* + PGBKT7–*IlMT2a*) were transformed into YH2GOLD yeast strain using the Yeastmaker™ Yeast Transformation System 2 (Takara). After growing in SD-Trp-Leu and SD-Trp-Leu/x-α-Gal medium (Takara) for 3 days, the monoclones were inoculated into YPDA liquid medium for 24 h of dark culture. Ten-fold serial dilutions (1, 1:10, 1:10^2^, 1:10^3^ and 1:10^4^) were spotted onto SD-Trp-Leu and SD-Trp-Leu /x-α-Gal solid media for 3 days to observe the phenotype.

The full-length ORF sequence of *IlAP2* was inserted into the PSM35S–NYFP vector using the ClonExpress II One Step Cloning Kit (Vazyme). The full-length *IlMT2a* ORF sequence was inserted into the PSM35S–CyFP vector to form the recombinant vector PSM35S–*IlMT2a*–CyFP. The constructed vector plasmids were transferred into *Agrobacterium tumefaciens GV3101* using electro-transformation method and cultured for 2 days at 30 °C. Subsequently, the constructed vector plasmids were mixed in a 1:1 ratio and injected into the lower epidermis of tobacco leaves. The results were observed with confocal laser scanning microscopy (Zeiss LSM 710 META) after 2 days under low light conditions with BRZ1-nTFP + HLH40-cYFP as positive control [47,48,49].

### 4.5. Construction and Stress Treatment of Transgenic A. thaliana

The *IlAP2* sequence was inserted into the pCAMBIA1305 vector with 35S promoter using the ClonExpress II One Step Cloning Kit (Vazyme), then the 35S:*IlAP2* plasmid was transformed into *A. tumefaciens GV3101*. *A. thaliana* Columbia was used for transformation by tidbit infection, and homozygous T3 transgenic *A. thaliana* lines were identified with 1/2 MS medium containing selective antibiotic hygromycin (50 mg/L). The expression of *I1AP2* in WT and transgenic *A. thaliana* was confirmed by agarose gel electrophoresis with PCR products amplified using IlAP2-ORF primers and cDNA from WT and transgenic *A. thaliana*. Total RNA from WT and transgenic *A. thaliana* under Cd stress was extracted using Plant RNeasy Mini Kit (Vazyme), and cDNA was generated using PrimeScript^®^ RT kit (Takara). Three-day-old WT and transgenic *A. thaliana* seedlings were selected and transplanted to 1/2 MS medium containing 50 μM CdCl_2_. The root length and fresh weight of seedlings were measured after 7 days of growth.

### 4.6. RNA-seq and Analysis

Twenty-day-old transgenic and WT *A. thaliana* seedlings were treated with 50 μM CdCl_2_ for 24 h and sampled for RNA extraction [50]. RNA-seq libraries were created following the manufacturer’s instructions for the NEBNext^®^ UltraTM RNA Library Prep Kit for Illumina^®^ (NEB, Ipswich, MA, USA). The cDNA fragments, ranging in length from 250 to 300 bp, were screened using AMPure XP system (Beckman Coulter, Beverly, CA, USA). Illumina Hiseq 2000 (Illumina, San Diego, CA, USA) was utilized for library sequencing and paired-end read production.

Genes were assembled using the full-length transcript sequence as a reference [51]. The FPKM value obtained from read counts was converted to a TPM value to obtain the expression level of each isoform. Based on analysis using differential expression analysis software DESeq2, genes with padj < 0.05 and |log_2_foldchange| > 1 were identified as differentially expressed genes (DEGs). GO enrichment analysis was performed on DEGs using Wallenius’ noncentral hypergeometric distribution of the GOseq R package. The KEGG enrichment analysis was performed using KOBAS software.

## 5. Conclusions

In conclusion, the AP2/ERF superfamily gene *IlAP2*, confirmed to interact with *IlMT2a* in this study, was analyzed using quantitative real-time PCR, transgenics and transcriptome sequencing to understand its regulation function and mechanism in improving plant tolerance to cadmium stress. The results showed that *IlAP2* could interact with IlMT2a in the nucleus and may cooperate with other transcription factors to regulate genes related to signal transduction and plant hormones, leading to reduced cadmium toxicity. These findings provide insights into the mechanism of *IlAP2*-mediated stress responses to cadmium and highlight *IlAP2* as an important gene resource for improving plant stress tolerance in phytoremediation to manage cadmium soil contamination.

## Figures and Tables

**Table 1:** Primer sequences of the genes for full-length open reading frames (ORFs) and semi-quantitative PCR and quantitative real-time PCR.

**Primer**	**Forward PCR Primer**	**Reverse PCR Primer**
IlAP2-ORF	ATGTCGTTCGACCTGAACTTC	TCAGCTCCTGGAGTGGTAATG
IlAP2-qRT-PCR (semi-quantitative PCR)	GAATTTGAGGATTTCTCAACC	TACTTGATGATGCTCAGGAAC

## Data Availability

No new data were created or analyzed in this study. Data sharing is not applicable to this article.

## Supplementary Materials

The following supporting information can be downloaded at: https://www.mdpi.com/article/10.3390/plants12040823/s1, Figure S1: Relative changes in expression of genes analyzed using qRT-PCR; Table S1: 1/2 Hoagland’s solution composition.
