# Supplementary material for: IlAP2, an AP2/ERF Superfamily Gene, Mediates Cadmium Tolerance by Interacting with IlMT2a in Iris lactea var. chinensis
Source: Plants (Basel). 2023 Feb 12;12(4):823. doi: 10.3390/plants12040823 (PMC9959467; doi:10.3390/plants12040823)
Supplement: Supplementary file 1 [file plants-12-00823-s001.zip › Table S1.pdf]

Table S1 1/2 Hoagland's solution composition

**Nutrient elements**	**Concentrations**
**Calcium Nitrate Tetrahydrate**	473
**Nitrate of Potash**	253
**Ammonium Nitrate**	40
**Potassium Dihydrogen Phosphate**	68
**Magnesium Sulfate**	247
**Iron Vitriol**	2780
**EDTA•Na**	3730
**Potassium Iodide**	415
**Boric Acid**	3.1
**Manganese Sulfate**	11.2
**Zinc Sulfate**	4.3
**Sodium Molybdate**	0.13
**Copper Sulfate**	0.013
**Cobalt Chloride**	0.013
