# Supplementary figures and images for: IlAP2, an AP2/ERF Superfamily Gene, Mediates Cadmium Tolerance by Interacting with IlMT2a in Iris lactea var. chinensis
Source: Plants (Basel). 2023 Feb 12;12(4):823. doi: 10.3390/plants12040823 (PMC9959467; doi:10.3390/plants12040823)

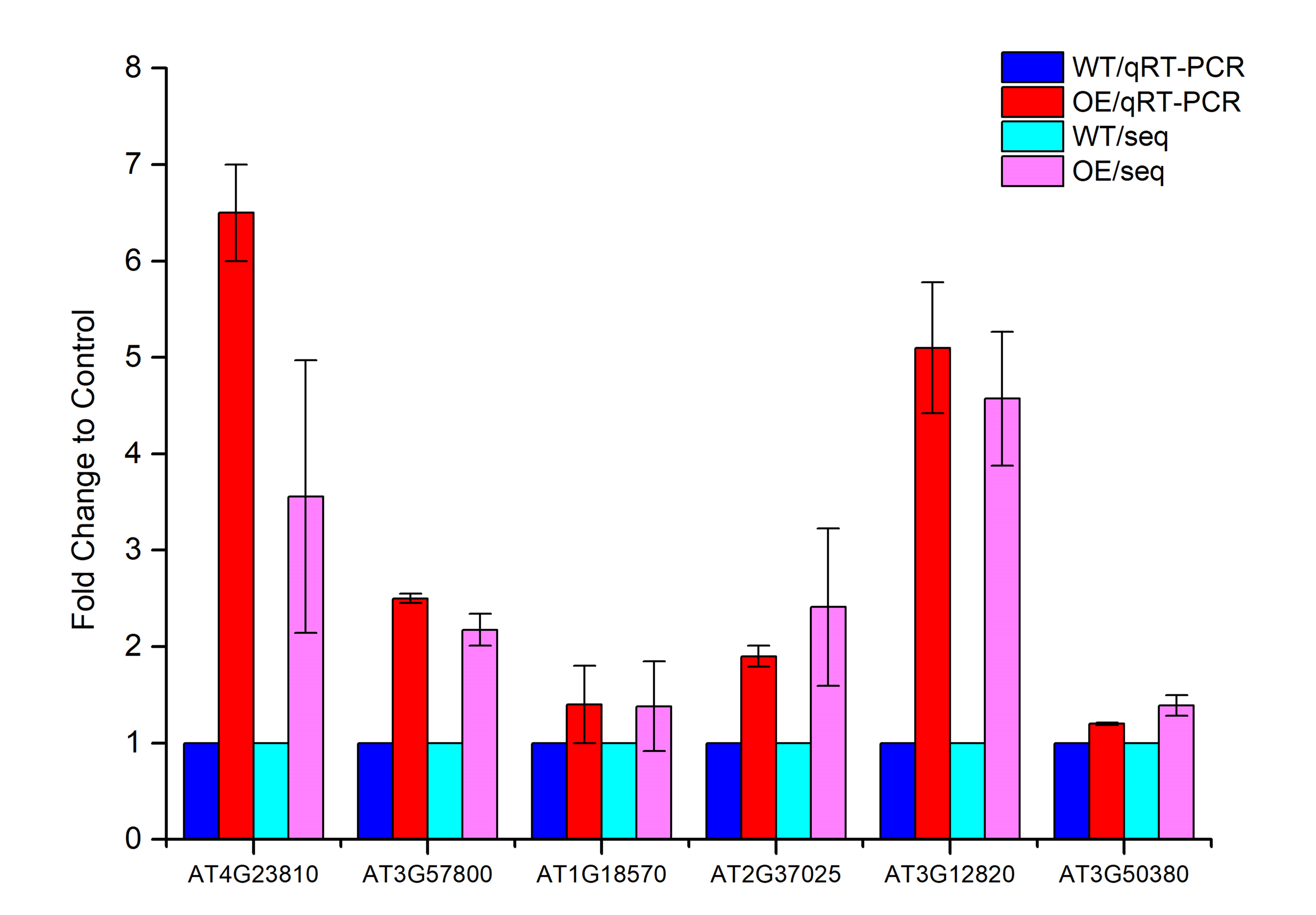

Supplement: Supplementary file 1 [file plants-12-00823-s001.zip › Figure S1.jpg]
